# A Learning Dendritic Neuron-Based Motion Direction Detective System and Its Application to Grayscale Images

**DOI:** 10.3390/brainsci14090864

**Published:** 2024-08-27

**Authors:** Tianqi Chen, Yuki Todo, Ryusei Takano, Zhiyu Qiu, Yuxiao Hua, Zheng Tang

**Affiliations:** 1 Division of Electrical Engineering and Computer Science, Kanazawa University, Kanazawa 920-1192, Japan; chentianqi@stu.kanazawa-u.ac.jp (T.C.); qiuzy1916@stu.kanazawa-u.ac.jp (Z.Q.); sh13818971028@gmail.com (Y.H.); 2 Faculty of Electrical, Information and Communication Engineering, Kanazawa University, Kanazawa 920-1192, Japan; 3 Kanazawa Engineering System Inc., Kanazawa 920-1158, Japan; takano296@kanazawa-es.com; 4 College of Information Science and Technology, Eastern Institude of Technology, No. 568, Tongxin Road, Ningbo 315200, China; 5 Faculty of Engineering, University of Toyama, Toyama 930-8555, Japan

**Keywords:** artificial visual system, neural network, dendritic neuron, motion direction detection, deep learning

## Abstract

In recent research, dendritic neuron-based models have shown promise in effectively learning and recognizing object motion direction within binary images. Leveraging the dendritic neuron structure and On–Off Response mechanism within the primary cortex, this approach has notably reduced learning time and costs compared to traditional neural networks. This paper advances the existing model by integrating bio-inspired components into a learnable dendritic neuron-based artificial visual system (AVS), specifically incorporating mechanisms from horizontal and bipolar cells. This enhancement enables the model to proficiently identify object motion directions in grayscale images, aligning its threshold with human-like perception. The enhanced model demonstrates superior efficiency in motion direction recognition, requiring less data (90% less than other deep models) and less time for training. Experimental findings highlight the model’s remarkable robustness, indicating significant potential for real-world applications. The integration of bio-inspired features not only enhances performance but also opens avenues for further exploration in neural network research. Notably, the application of this model to realistic object recognition yields convincing accuracy at nearly 100%, underscoring its practical utility.

## 1. Introduction

Building bio-inspired neural network models is regarded as a promising approach for solving various computer vision problems, such as orientation detection, object detection, and motion direction [1]. These bio-inspired models are applied in numerous technologies. However, simulating the brain’s complex neural network, which comprises over $10^{11}$ neurons and more than $10^{15}$ interactions, presents significant challenges [2]. Approximately 80% of sensory information received by the brain is vision-based, with the retina processing more than half of this visual information [3,4]. The visual data from the outside world, including forms, colors, orientations, and movements, significantly influence human actions and decisions. Among this visual information, the perception of objects and the detection of their movement direction are particularly crucial, as they are directly linked to survival [5]. Therefore, understanding the methods and mechanisms of motion direction detection in vision is a key research theme for elucidating the structure and function of the visual cortex [6]. Research in this area has been ongoing for decades, leading to the proposal of various models aimed at explaining how the brain processes motion direction [7,8]. This research is critical for advancing our understanding of the visual cortex and its role in interpreting and responding to visual stimuli. Hubel and Wiesel implemented a series of experiments on the visual cortex of animals and recorded many significant biological phenomena, contributing greatly to the research of the visual system [9]. They found that some neurons only respond to light stimulation in a specific orientation and are insensitive to the position of the stimuli [10]. The movement of the optimally oriented light stimulation within their receptive fields does not cause neuron inactivation. Bio-inspired models are based on these mechanisms [11]. However, since the specific mechanism of the visual cortex is unclear, most explanations of the visual mechanism so far have been primarily motivated by speculation and non-quantitative methods. Bio-inspired models are based on these mechanisms, even though most explanations of the visual mechanism so far have been primarily motivated by speculation and non-quantitative methods. Based on the results of vision experiments on insects such as flies, Hassenstein and Reichardt proposed a neuron model in 1956 that detects the direction of motion by two spatially different inputs that take into account the time difference [12]. In the same year, the Barlow–Levick model was proposed to explain the results of experiments on rabbit retinal ganglion cells [13]. In 1967, 102 types of direction-selective cells were identified in rabbit retinas [14], and similarly selective cells were found in the retinas of mammals such as humans [15,16]. In 1982, Torre, Poggio, and colleagues proposed a neuron model based on the results of experiments on cat retinal ganglion cells [17]. This model takes into account dendritic processing and has the function of selecting the direction of motion due to the nonlinear interaction of synaptic connections on the dendrites and the time difference of the inputs. This is based on the assumption that the reaction of motion direction selectivity is determined by the synaptic connections on the dendrites [18]. It is also believed that the ability to detect the direction of motion is not innate but is influenced by the external environment after birth instead of before. If sufficient visual stimulation is not provided, visual impairment can result. One example is a study by Blakemore and Cooper in 1970, which revealed that cats exposed only to vertical stimulation after birth did not respond to horizontal stimulation. This suggests that the ability to detect the direction of motion is gradually acquired through exposure to visual stimulation after birth [19].

Recent research on motion direction detection of objects has seen advancements in various domains, particularly in neuromorphic sensors, object tracking algorithms, and artificial visual systems. A significant development is a neuromorphic visual sensor designed to recognize moving objects and predict their trajectories. This sensor mimics biological vision, using spiking neural networks to process visual information in a way that is both energy-efficient and effective at predicting motion paths in real time. This technology has promising applications in robotics and autonomous systems [20]. Researchers have also been working on improving object-tracking algorithms by combining motion direction data with time-series information. A new algorithm introduced in 2023 enhances the accuracy of object localization in video sequences by incorporating a mechanism that evaluates the reliability of tracking results. This approach significantly improves performance, particularly in challenging scenarios like object occlusion and varying illumination [21]. In 2023, we developed a novel artificial visual system inspired by retinal direction-selective pathways. This system mimics how certain ganglion cells in the retina detect motion directions. The AVS model was tested extensively, demonstrating superior performance in detecting motion directions, even under noisy conditions. It also showed advantages in hardware implementation and learning complexity when compared to state-of-the-art deep learning models [22].

This research aims to utilize a dendritic neuron model for simulating motion direction detection processing in the primary visual cortex. The dendritic neuron model extends existing biologically plausible models, which have been shown to be highly efficient in data processing and accurate in prediction and regression tasks involving one-dimensional inputs [23,24]. Previously, these models demonstrated remarkable capabilities in capturing complex neural computations and accurately modeling neuronal responses to one-dimensional stimuli. However, their application to two-dimensional image processing tasks, particularly motion detection, has not been fully explored. To detect motion direction in two-dimensional images, the authors designed a model capable of high-accuracy detection in black and white binary images [25]. This model was a significant step forward in utilizing dendritic neuron models for visual tasks, showing that these models could be adapted for more complex visual processing. However, this model limited application to only binary images. In this study, we aim to address these limitations by integrating horizontal cellular mechanisms into the dendritic neuron model, thereby enhancing its ability to process grayscale images. Horizontal cells are crucial in visual processing due to their center–surround receptive fields, which enhance contrast and modulate communication between photoreceptor cells and bipolar cells [26]. These cells play a vital role in the initial stages of visual information processing by integrating and regulating input from multiple photoreceptors, thereby contributing to the formation of the visual signal transmitted to the brain. They are responsible for transmitting visual signals to the ganglion cells and eventually to the brain [27,28]. The On–Off Response mechanism of horizontal cells enables the detection of differences between images before and after motion, which is essential for accurate motion detection. Since dendrites are deeply involved in neuron function and selectivity, this study proposes a motion direction detection model using the dendritic neuron model. The new model incorporates the role of horizontal cells to enhance motion detection accuracy. By simulating the center–surround receptive fields and the On–Off Response mechanisms of horizontal cells, the model aims to more accurately replicate the biological processes of the primary visual cortex. To address the limitations of previous models, this study introduces an artificial visual system (AVS) capable of detecting motion direction with high accuracy even in grayscale images, extending beyond the initial focus on black and white images. The AVS integrates advanced features of dendritic processing and horizontal cell function to improve both the efficiency and accuracy of motion direction detection. The results show that this model is more effective in detecting motion direction than general CNNs, achieving higher accuracy and efficiency by leveraging the detailed biological mechanisms of visual processing. This enhanced approach not only provides a more accurate simulation of visual processing in the primary visual cortex but also sets the stage for future research in applying dendritic neuron models to other complex visual tasks. The incorporation of horizontal cell dynamics represents a significant conceptual advancement, bridging the gap between biological plausibility and computational efficiency in motion detection models.

In this article, we first introduce the dendritic model, followed by the integration of horizontal and bipolar cells’ On–Off Response mechanisms into the dendritic neuron model. Based on this, a local motion direction detective dendritic neuron model is designed, which is then extended to a global motion direction detective dendritic neuron model. A learning mechanism utilizing backpropagation is also discussed. The article concludes with comparisons across various CNN models, including cross-comparisons using different datasets as training sets, overall dataset comparisons, and comparisons of successfully trained models on real-world datasets and rotated MNIST. Finally, we summarize the conclusions of our work and discuss the strengths and weaknesses of our model.

## 2. Method

### 2.1. Dendritic Neuron Model

The McCulloch–Pitts model laid the foundation for modern artificial neural networks and their variations [29]. The dendritic neuron model, validated for its biological alignment, leverages dendritic functionality crucial for computer vision tasks [30]. Unlike the McCulloch–Pitts model, which ignored the role of dendrites, it is now understood that dendrites play a crucial role in motion detection [31]. Recent research has revealed the existence of nonlinear interactions between synaptic connections, suggesting that even individual neurons can perform complex calculations [32]. These advanced computational capabilities are believed to be realized through interactions between synapses on dendrites, the function of dendrites as independent subunits, and the sophisticated computational network capabilities of individual neurons [33]. Therefore, the dendritic neuron model can be viewed as operating in a manner consistent with the models proposed by Koch, Poggio, and Torre [17]. This research employs a dendritic neuron model that emphasizes neural processing not only in the soma and axon but also in dendrites [17,34]. This dendritic neuron model consists of four layers—synaptic, branches, membrane, and soma—each with distinct functions rooted in biology.

**Figure 1:**
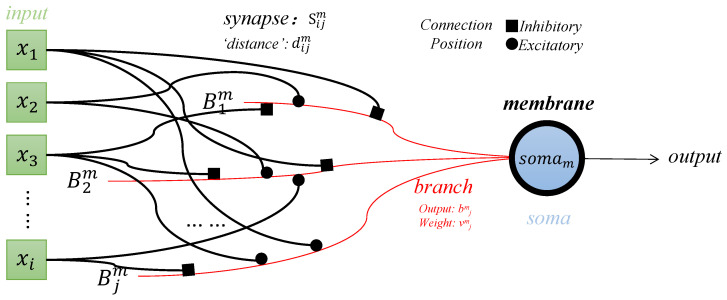
A sample structure of the dendritic neuron model.

Figure 1 illustrates the dendritic neuron model, which comprises a set of independent branches $\left(B_{j},j=1,2,\ldots ,m\right)$ (red curves) and a soma (blue circle) with its membrane (black bold curve). A set of inputs $x_{1},x_{2},\ldots ,x_{n}$ is applied to the branches of the neuron through their own synapses (black curves). The signals, post-synaptic processing, are multiplied at each branch, and these products are weighted by branch strength and summed to determine the neuron’s activation level.

1. **Synaptic layer:** This layer integrates inputs from other neurons and transforms them into a single output $S_{ij}$ using a sigmoid function. For a dendritic neuron model with *i* inputs and *j* branches, the synaptic function is given by:
  (1) $$S_{ij}\left(x_{i}\right)=\frac{1}{1+exp(-\frac{1}{d_{ij}}\left(w_{ij}x_{i}-q_{ij}\right))}.$$
  This step endows dendritic neurons with learning capabilities. The $d_{ij}$ is the distance parameter of the input, and $w_{ij}$, $q_{ij}$ are the weight and bias of the synapse. Through a learning algorithm, the model can prune ineffective synapses and branches by the iteration of weights and bias, enhancing detection accuracy.
2. **Branch layer:** This layer distributes inputs to the soma layer through the function [19]:
  (2) $$b_{j}=\prod_{i=1}^{N}S_{ij}\left(x_{i}\right).$$
  where *N* is the number of synapses. The output is entirely influenced by the synaptic output, resulting from the multiplication of inputs. A constant-1 synapse does not alter the output, while a constant-0 synapse nullifies it, which is essential for the learning feature of the dendritic model.
3. **Membrane layer:** This layer, representing the dendritic neuron’s plasma membrane, regulates ion flow through voltage-gated ion channels. The membrane potential changes in response to synaptic inputs and is integral to the neuron’s ability to generate action potentials. This layer represents the neuron’s ability to process integrated signals and prepare for signal transmission. In real neurons, the membrane potential is influenced by the sum of excitatory and inhibitory inputs, leading to changes in the neuron’s overall activity. The function is expressed as:
  (3) $$u=\sum_{j=1}^{M}v_{j}b_{j}.$$
  where *M* is the number of branches. Here, information is aggregated by summing the inputs $b_{j}$ with connection strength $v_{j}$ and then processed by the soma.
4. **Soma layer:** The cell body performs nonlinear processing of the integrated inputs from the branch layer, combining them to produce a single output. This is expressed by:
  (4) $$O=\frac{1}{1+exp(-\lambda (u-\theta ))}.$$
  where θ is its own threshold and λ is the slope parameter of the soma.

**Figure 2:**
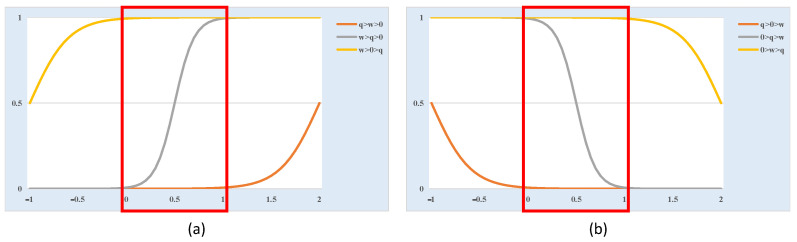
Sigmoid function behavior under different synaptic weights, with the red box indicating the accepted signal range (0 to 1).

As mentioned above, the dendritic neuron model is a feed-forward network and all units are differentiable. Thus, a traditional error backpropagation algorithm can be used to train the neuron by adjusting its synaptic weights $w_{ij}^{m}$, and bias $q_{ij}^{m}$. As shown in Figure 2, due to the constraints of the neural signal range, only the input signal between 0 and 1 is considered. Several scenarios should be evaluated due to the intricate relationship among connection positions and the interplay among $w_{ij}^{m}$ and $q_{ij}^{m}$.

For $w_{ij}^{m}>0$ in Figure 2a, the sigmoid function exhibits an upward trend. When $q_{ij}^{m}>w_{ij}^{m}>0$, the function’s output remains within the range of 0, indicating a constant-0 state. Due to the dendritic neuron’s nonlinear multiplication algorithm, the entire branch is effectively severed. Similarly, when $w_{ij}^{m}>q_{ij}^{m}>0$, the function’s output is consistently 1, indicating a constant-1 state, rendering the input at this synapse insignificant. However, when $w_{ij}^{m}>q_{ij}^{m}>0$, the function output varies with the input, putting the synapse in an excitatory state. For $w_{ij}^{m}<0$ in Figure 2b, the sigmoid function shows a downward trend. When $q_{ij}^{m}>0>w_{ij}^{m}$, the synapse is in a constant-0 state. When $0>w_{ij}^{m}>q_{ij}^{m}$, the synapse is in a constant-1 state. However, when $0>q_{ij}^{m}>w_{ij}^{m}$, the function output varies with the input, putting the synapse in an inhibitory state.

It is precisely through this customizable functionality that we have endowed the dendritic neuron model with the capability of motion direction detection. By fine-tuning synaptic weights and biases, the model can effectively learn to detect motion direction, demonstrating its versatility and learning ability in processing complex visual information.

### 2.2. Local Motion Direction Detective Dendritic Neuron Model

**Figure 3:**
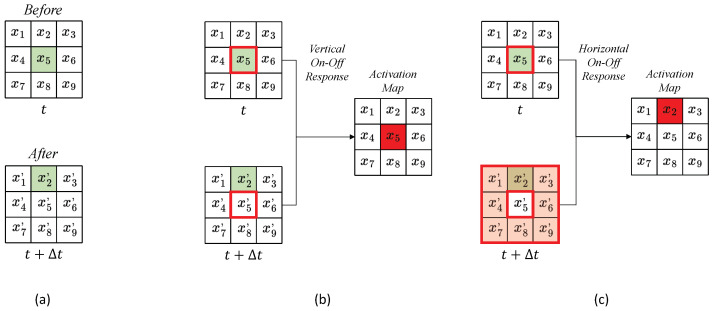
An example of horizontal and vertical On–Off Response mechanisms: (**a**) is the signal of 3 × 3 photoreceptors of the images before and after movement, (**b**) is the activation of the vertical On–Off Response mechanism, and (**c**) is the activation of the horizontal On–Off Response mechanism, where the red boxes indicate the corresponding neurons being activated. White squares represent photoreceptors with no light input, while green squares represent those with received input.

The detection of the direction of motion of an object begins when the retina receives light stimuli from the external environment. The light stimuli include information about the object’s brightness, darkness, and spatiotemporal color changes. The retina captures this information and converts it into electrical signals in the photoreceptor cells. These signals are then transmitted to the bipolar cells, where they are processed based on light intensity. This processing is relayed directly to the ganglion cells via the excitatory vertical pathway. Since ganglion cells also receive inputs from horizontal cells, they indirectly receive signals from surrounding cells. Horizontal cells send inhibitory inputs horizontally in response to the vertical excitatory pathway. Retinal ganglion cells exhibit motion direction selectivity, responding strongly to stimuli moving in a specific direction but not at all to stimuli moving in the opposite direction [8]. On–Off motion direction selective ganglion cells have also been identified. There are On-centered ganglion cells that respond to bright light in the center of their receptive field and Off-centered ganglion cells that respond to dim light in the center. The periphery of the On-centered field responds to dim light, while the periphery of the Off-centered field responds to bright light [35]. Since dendritic shapes are acquired through later training, the connection state must be determined by learning. To address these challenges, we propose an AVS that can detect the direction of object movement for objects of any size, shape, and position in two-dimensional grayscale images. This system passively adjusts the dendrites using the dendritic neuron model, which can acquire motion direction selectivity through learning [36]. Furthermore, to handle grayscale images, we employed an On–Off Response mechanism that incorporates the processing of horizontal and bipolar cells, which includes both horizontal and vertical On–Off Response mechanisms. Both types of On–Off Response mechanisms require a threshold to function effectively. Figure 3a shows a 1-pixel object moving above a 3×3 receptive field. We designed a receptive field of 3×3 pixels, representing 9 photoreceptors. The photoreceptors receive stimuli from the image, simulating the process occurring in the retina. The image before movement at time *t* and the image after moving at time t+Δt are regarded as the pre-movement and post-movement inputs. We denote the pixels in the pre-movement image as $x_{1},x_{2},\cdots ,x_{9}$ and the pixels in post-movement with $x_{1}^{\prime },x_{2}^{\prime },\cdots ,x_{9}^{\prime }$. In Figure 3b, the activation map and the corresponding pixels of the vertical On–Off Response mechanism during movement are shown. This mechanism compares the center of the images before and after movement and reacts to changes in brightness, a process occurring in the bipolar cells. The vertical On–Off Response mechanism compares the center pixels, $x_{5}$ and $x_{5}^{\prime }$, with the vertical threshold $\epsilon_{V}$, using the following equation:

(5) $$BC=\left\{\begin{array}{c} 1,(|x_{5}-x_{5}^{\prime }|>\epsilon_{V})\\ 0,(|x_{5}-x_{5}^{\prime }|<\epsilon_{V}) \end{array}\right.$$

As a result, when the center pixel has an obvious change in grayscale value, the bipolar cell is activated. Figure 3c illustrates the horizontal On–Off Response, which inhibits areas where there is a change in grayscale value at the center of the pre-movement image and the periphery of the post-movement image. It does not respond in areas with no change in grayscale value. This type of horizontal On–Off Response occurs in the horizontal cells. Similarly, the horizontal On–Off Response mechanism uses horizontal cells to compare the post-movement surrounding pixels $x_{1}^{\prime },\cdots ,x_{4}^{\prime },x_{6}^{\prime },\cdots ,x_{9}^{\prime }$ with the pre-movement center pixel $x_{5}$:

(6) $$HC_{i}\left(i\neq 5\right)=\left\{\begin{array}{c} 1,(|x_{5}-x_{i}^{\prime }|<\epsilon_{H})\\ 0,(|x_{5}-x_{i}^{\prime }|>\epsilon_{H}) \end{array}\right.$$

Here, $\epsilon_{H}$ is the threshold of the horizontal On–Off Response mechanism. These thresholds enable the model to accurately detect changes in brightness and contrast, essential for motion direction detection in the dendritic neuron model.

**Figure 4:**
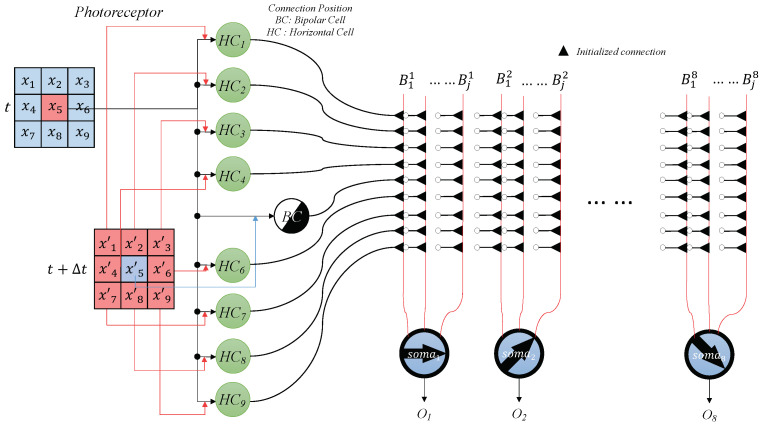
The initial structure of a group of local motion direction detective dendritic neurons that have 8 directions. Red squares represent inputs to the dendritic model, red curves indicate the branches of the dendritic model, and triangles mark the initial connection states.

Leveraging these two types of cells, dendritic neurons can detect receptive fields within a group of pixels. We integrate this into a local motion direction-detecting dendritic neuron model. As described earlier, the outputs of bipolar and horizontal cells serve as the inputs for the dendritic neurons. Figure 4 illustrates the structure of these local motion direction-detecting dendritic neurons. The inputs, which are the outputs from bipolar and horizontal cells, connect to the *j* branches of one of the local motion direction-detecting dendritic neurons. Each neuron is specialized for one of eight directions: up-left (135°), up (90°), up-right (45°), left (180°), right (0°), down-left (225°), down (270°), and down-right (315°). Initially, all synapses connect with the branches at undetermined positions (black triangles).

For clearer statements, the white circles are connected to the outputs of the horizontal cells and bipolar cells.

We use $x_{i}$ to represent the outputs of the On–Off Response mechanism, since the functions of the On–Off Response mechanisms we used are non-differentiable piecewise functions, and $x_{ijm}^{yz}$ is the center pixel of a receptive field located in the (y,z) of an image:

(7) $$x_{ijm}^{yz}=\left\{\begin{array}{c} HC_{i},\left(i\neq 5\right)\\ BC_{i},\left(i=5\right) \end{array}\right..$$

The reactions in the synapses in a local motion direction detective dendritic neuron for a center pixel $x_{5}$, which connects the input of *i*th pixel to the *j*th branch in $soma_{m}$, can be expressed by the following formula:

(8) $$S_{ijm}^{yz}\left(x_{ijm}^{yz}\right)=\frac{1}{1+\exp(-\frac{1}{d_{ijm}}\left(w_{ijm}x_{ijm}^{yz}-q_{ijm}\right))},$$

The results are then passed into branches and computed by calculating:

(9) $$b_{jm}^{yz}=\prod_{d=1}^{9}S_{ijm}^{yz}\left(x_{ijm}^{yz}\right).$$

Next, the branch outputs are aggregated at the membrane layer:

(10) $$u_{m}^{yz}=\sum_{j=1}^{M}v_{j}b_{jm}^{yz}.$$

Finally, the aggregated signals are processed in the soma to determine the corresponding motion direction:

(11) $$O_{m}^{yz}=\frac{1}{1+\exp(-\lambda \left(u_{m}^{yz}-\theta_{m}\right))}$$

These calculations enable the dendritic neuron model to effectively detect the direction of motion in local receptive fields, enhancing its utility in visual processing tasks.

### 2.3. Global Motion Direction Detective Dendritic Neuron Model

**Figure 5:**
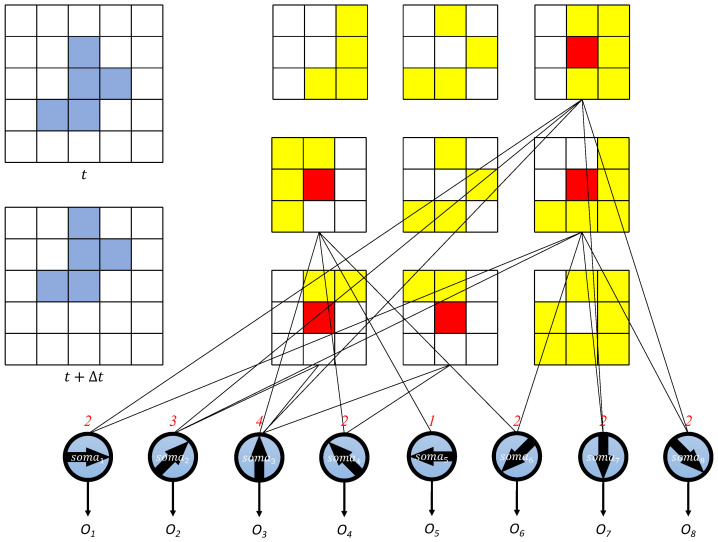
The global motion direction detective dendritic neurons and their application on a 5 × 5 up-direction image.

Building upon the local motion direction detective dendritic neuron model, we can extend this approach to analyze global motion direction across an entire image. This is achieved by examining each receptive field in the image and determining the most frequently selected direction of motion from the local models. The process involves applying the local motion direction detective dendritic neuron model to each receptive field within the image. Each receptive field provides an output indicating its detected motion direction. The global motion direction is then determined by aggregating the outputs of all receptive fields and selecting the direction that occurs most frequently. Here, we utilize a 5×5-pixel image to demonstrate the mechanism of the global motion direction detection model in Figure 5. In the figure, the 5×5 image can be divided into 3×3 local motion direction detective neurons that each have 3×3 receptive fields. In the pre-movement image *t* and post-movement image t+Δt, the object is colored blue and the background is white. By processing the vertical and horizontal On–Off Response mechanisms in bipolar cells and horizontal cells, the activation positions of local motion direction detective neurons are presented. In the activation map, the red squares show the excitatory responses of the bipolar cells, while the yellow squares indicate the excitatory responses of the horizontal cells. Other areas show no activation in the corresponding pixels. The results of the local motion direction-detective neurons are then aggregated in the soma, represented by the red numbers above the soma. This aggregation allows the model to determine the overall direction of motion across the image by selecting the most frequently activated direction from the local models.

The figure illustrates how the local motion direction-detecting dendritic neurons are applied to a 5×5-pixel image, analyzing each receptive field independently to determine the overall motion direction. The detection result of each direction can be regarded as the following equation:

(12) $$D_{m}=\sum_{y=1}^{M=5}\sum_{z=1}^{N=5}O_{m}^{yz}.$$

Figure 5 obviously shows that the ‘up’ direction has the most activation after (12), which has the same result as the observation of humans. By systematically applying the local models to each part of the image and aggregating the results, the global model effectively identifies the predominant direction of motion. This method leverages the detailed analysis provided by local models and combines it to form a comprehensive understanding of motion across the entire image.

### 2.4. Learning Algorithm

**Figure 6:**
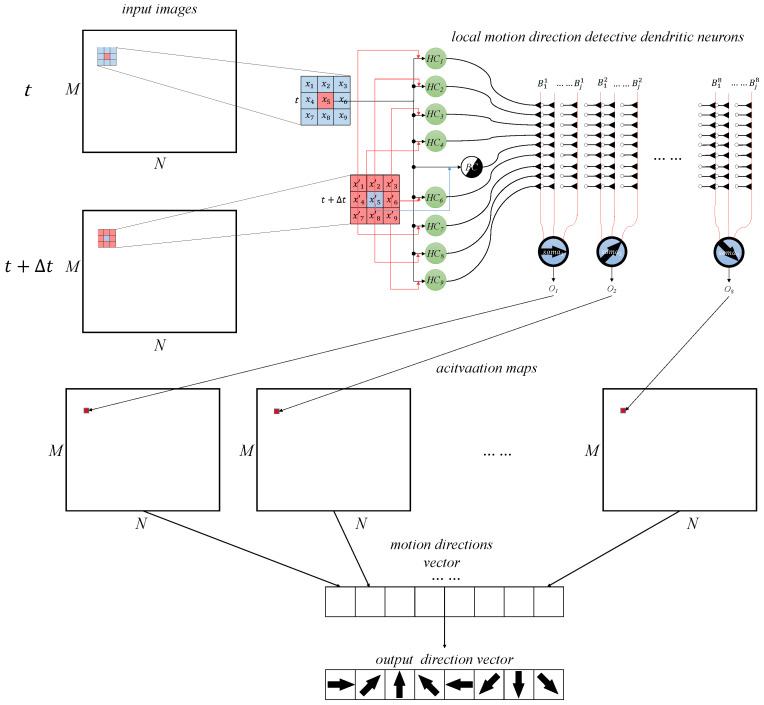
The structure of the AVS.

Our motion direction detective dendritic neuron is designed to learn and adapt based on input stimuli. The theory proposed by Blakemore suggests that motion direction selectivity develops due to external factors from birth, a notion supported by contemporary research [19,37]. Consequently, the synaptic connection states of the dendrites must be determined through learning. To facilitate this, we implement a learning function using the cross-entropy loss function, expressed as:

(13) $$E=-\sum_{m=1}^{8}T_{m}\log\left(\bar{D_{m}}\right)$$

where *E* is the cross-entropy loss, $\bar{D_{m}}$ is the normalized overall output for motion direction detection in a group of M×N input images, and $T_{m}$ presents the teacher’s signal for the motion direction detection. Given that the AVS architecture for global motion direction detection functions as a feed-forward network, the motion direction detective neurons can be trained using the error backpropagation method. By employing this approach, we endow the system with the ability to learn. The structure of the system is illustrated in Figure 6.

In the figure, a set of single-channel data images of size M×N for the pre-movement image at time *t* and the post-movement image at time t+Δt are scanned by the local motion direction detective dendritic neuron to compose the global motion direction detective system and produce the activation map. Based on the activation map, the detection result is output by the system. These results are then used in the learning system to improve accuracy and performance over time.

In the overall architecture of our AVS, the loss function is influenced by several parameters, which are essential for the backpropagation learning algorithm. We have made the weights $w_{ijm}$ and biases $q_{ijm}$ of each dendritic neuron learnable, allowing these parameters to be adjusted during training. Meanwhile, other parameters, such as the distance parameter $d_{ij}$ and branch constants, are fixed. This simplification is based on the premise that while these parameters contribute to the neuron’s function, their variation adds unnecessary complexity to the model, making computational calculations more demanding. The On–Off Response mechanisms in the visual system, which help differentiate light and dark contrasts, have a significant genetic basis. Research has shown that these mechanisms are largely determined by the intrinsic properties of retinal cells and their genetic programming [38]. The separation of On and Off signals begins at the photoreceptor level and is further processed by bipolar and ganglion cells in the retina. These processes are genetically encoded and are essential for basic visual function from birth [39]. As a result, the thresholds of the bipolar cells and horizontal cells are also fixed constants. We change the values of the synaptic connection parameters $w_{ijm}$ and $q_{ijm}$ so that the error *E* is reduced with

(14) $$\Delta w_{ijm}=-\eta \frac{\partial E}{\partial w_{ijm}},$$

(15) $$\Delta q_{ijm}=-\eta \frac{\partial E}{\partial q_{ijm}},$$

where η is the learning rate. Further, using the chain rule gives the following:

(16) $$\frac{\partial E}{\partial w_{ijm}}=\frac{\partial E}{\partial \bar{D_{m}}}\cdot \frac{\partial \bar{D_{m}}}{\partial D_{m}}\cdot \frac{\partial D_{m}}{\partial O_{m}}\cdot \frac{\partial O_{m}}{\partial b_{jm}}\cdot \frac{\partial b_{jm}}{\partial S_{ijm}}\cdot \frac{\partial S_{ijm}}{\partial w_{ijm}},$$

(17) $$\frac{\partial E}{\partial q_{ijm}}=\frac{\partial E}{\partial \bar{D_{m}}}\cdot \frac{\partial \bar{D_{m}}}{\partial D_{m}}\cdot \frac{\partial D_{m}}{\partial O_{m}}\cdot \frac{\partial O_{m}}{\partial b_{jm}}\cdot \frac{\partial b_{jm}}{\partial S_{ijm}}\cdot \frac{\partial S_{ijm}}{\partial q_{ijm}},$$

(18) $$\frac{\partial E}{\partial \bar{D_{m}}}=-\frac{T_{m}}{\bar{D_{m}}},$$

(19) $$\frac{\partial \bar{D_{m}}}{\partial D_{m}}\cdot \frac{\partial D_{m}}{\partial O_{m}}=MN\cdot \frac{1}{MN}=1,$$

(20) $$\frac{\partial O_{m}}{\partial b_{jm}}=\frac{v_{j}\lambda \exp\left(-\lambda \left(v_{j}-\theta_{m}\right)\right)}{{\left(1+\exp\left(-\lambda \left(v_{j}-\theta_{m}\right)\right)\right)}^{2}},$$

(21) $$\frac{\partial b_{jm}}{\partial S_{ijm}}=\prod_{a\neq i}S_{ajm},$$

(22) $$\frac{\partial S_{ijm}}{\partial w_{ijm}}=\frac{x_{ijm}\exp\left(-\frac{1}{d_{ijm}}\left(w_{ijm}x_{ijm}-q_{ijm}\right)\right)}{d_{ijm}{\left(1+\exp\left(-\frac{1}{d_{ijm}}\left(w_{ijm}x_{ijm}-q_{ijm}\right)\right)\right)}^{2}},$$

(23) $$\frac{\partial S_{ijm}}{\partial q_{ijm}}=-\frac{\exp\left(-\frac{1}{d_{ijm}}\left(w_{ijm}x_{ijm}-q_{ijm}\right)\right)}{d_{ijm}{\left(1+\exp\left(-\frac{1}{d_{ijm}}\left(w_{ijm}x_{ijm}-q_{ijm}\right)\right)\right)}^{2}}.$$

We update the synaptic parameters in a way that minimizes the error *E* until the error is minimized.

This structure forms the basis of our learnable motion direction detection AVS, which leverages the capabilities of dendritic neurons to accurately and adaptively detect motion direction through a trained system.

## 3. Simulation Results

### 3.1. Dataset Description

The dataset used in this study consists of grayscale single-channel images, each with a resolution of 32×32 pixels. Each image contains two main components: a static background and a moving object. The object moves randomly to one of its eight nearest pixels and is labeled according to the direction of movement. The motion direction is up-left (135°), up (90°), up-right (45°), left (180°), right (0°), down-left (225°), down (270°), or down-right (315°)

The objects vary in size, with eight different pixel dimensions: 1, 2, 4, 8, 16, 32, 64, and 128 pixels. The objects’ colors are also varied, including three types: completely random, solid colors ranging from 1 to 255, and black (0). Correspondingly, the backgrounds are assigned three types of colors: random, solid, and black. This results in eight combinations (black background with black object is excluded due to lack of contrast; abbreviations are used in the following tables):

(1) Black background with solid-colored object (dark background and light object, DL);
(2) Black background with random-colored object (dark background and random object, DR);
(3) Solid background with black object (light background and dark object, LD);
(4) Solid background with solid-colored object (light background and light object, LL);
(5) Solid background with random-colored object (light background and random object, LR);
(6) Random background with black object (random background and dark object, RD);
(7) Random background with solid-colored object (random background and light object, RL);
(8) Random background with random-colored object (random background and random object, RR).

**Figure 7:**
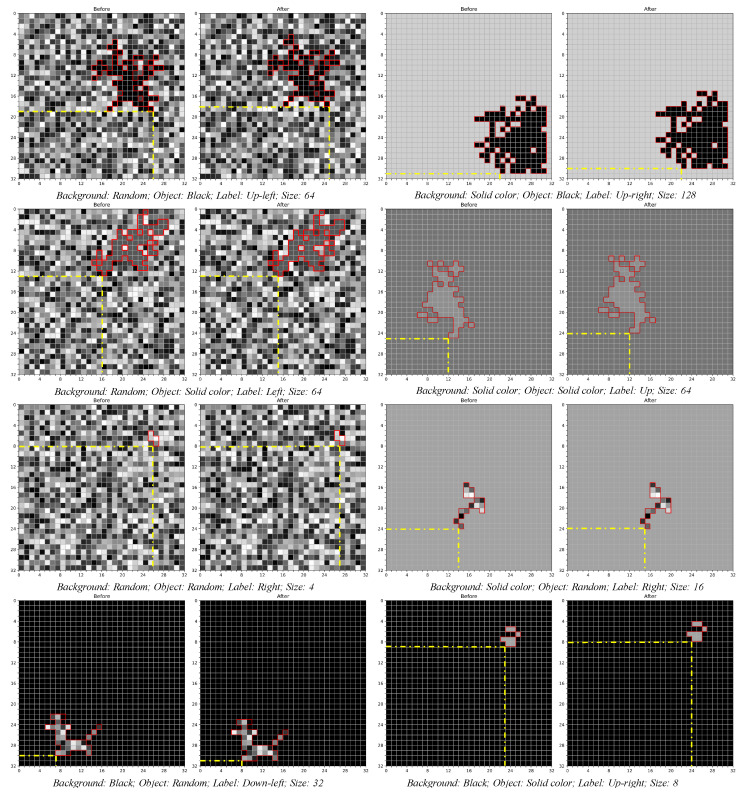
Samples of dataset images.

Each combination of object size and color has over 20,000 images. We randomly selected 10,000 images from each combination, splitting them into training and testing sets to evaluate the performance of our model and other SOTA (state-of-the-art) models. Examples of each image type are shown in Figure 7. This comprehensive dataset ensures a robust evaluation of the models’ capabilities in detecting motion direction across various object sizes and color configurations.

For simple models, such as basic or early CNN models, we applied random padding of size 1 to each channel of images. This technique mitigates the impact of edge artifacts on feature extraction, particularly in the presence of background noise. Additionally, this approach helps simulate the influence of edge values on subsequent motion occurring near the image boundaries. For more complex models, such as ResNet or EfficientNet, which are designed for three-channel inputs and can adapt to various input sizes, we padded the third channel with zeros. This approach ensures that no extraneous information is introduced, maintaining the integrity of the two-channel grayscale input data.

### 3.2. SOTA Model Description

In our study, we employ grayscale images in pairs to detect motion direction using a neural network that processes two-channel images. This approach, based on supervised learning with simple labels, lends itself to image classification networks rather than object detection networks like YOLO. Object detection models, while powerful, are designed for identifying and localizing multiple objects within an image rather than discerning motion direction from two frames [40]. Additionally, object detection models often involve more complex preprocessing steps and require more computational resources, which are not aligned with the simplicity and efficiency goals of our research [41].

We use the following simplified models:

**Four-Layer CNN:** We compare our model with a Four-Layer CNN due to its straightforward structure and ease of understanding. This model retains the core advantages of CNNs, such as automatic feature extraction and hierarchical feature learning, while minimizing computational complexity. These features make it suitable for benchmarking our motion direction detection model.
**LeNet:** As one of the earliest deep learning architectures, LeNet offers a balance between simplicity and performance. It extends beyond basic CNNs by introducing a wider application scope and higher accuracy. Its structured approach to convolution and pooling layers makes it a valuable baseline for comparison [42].

For deeper structural models, because our model is based on biological theory, it has a higher accuracy in image processing, so it can be compared with models with deeper structures based on accuracy and cross-validation like the following models:

**ResNet:** ResNet50 is known for its residual connections, which allow for the training of very deep networks by addressing the vanishing gradient problem. The ResNet50 model, with its 50 layers, excels in handling labeled image datasets, making it highly accurate and adaptable to our two-frame grayscale motion detection task. ResNet’s architecture includes residual blocks that facilitate gradient flow, improving convergence and accuracy. The key components include convolutional layers with batch normalization and ReLU activations, along with identity and convolutional shortcuts to form residual blocks, enhancing model performance without significantly increasing complexity [43].
**EfficientNet:** Designed for scaling model size efficiently, EfficientNet-B0 provides a balance between computational cost and accuracy. This model is particularly suitable for smaller datasets like ours, as it optimizes learning efficiency while maintaining robust performance. EfficientNet uses a compound scaling method that uniformly scales network depth, width, and resolution, enhancing both efficiency and accuracy. Its architecture includes a series of mobile inverted bottleneck MBConv blocks and squeeze-and-excitation optimization to improve feature representation while being computationally efficient [44].
**ConvNeXt:** ConvNeXt represents a modern approach to image classification with deep neural networks. Known for its high accuracy, this model leverages advanced techniques to enhance feature extraction and model training, making it an ideal candidate for validating our proposed model’s performance. ConvNeXt improves upon traditional CNNs by incorporating insights from transformer models, resulting in enhanced feature representation and improved classification accuracy. It employs a hierarchical architecture with stage-wise downsampling and a series of convolutional layers, offering a balance between spatial resolution and computational efficiency [45].

By comparing our learning dendritic neuron-based motion direction detection AVS with these state-of-the-art models, we aim to demonstrate its efficacy and potential advantages in terms of biological plausibility, learning efficiency, and robustness to noise. In our experiments, we determine the number of training epochs by ensuring that after obtaining initial training results, further training continues until both the training accuracy and test accuracy fluctuate by no more than 0.05% within the next 100 epochs, with the total fluctuation not exceeding 0.1%. This method ensures model stability and convergence, avoiding overfitting and underfitting and confirming that the model has reached a steady state. Such an approach is vital for reliable performance assessment and is supported by the best practices in machine learning research [46,47]. Notably, EfficientNet’s pre-training on ImageNet introduces an additional variable, rendering its results relatively less fair in this specific context.

### 3.3. Cross-Validation Accuracy

To rigorously evaluate the generalization capability of the proposed model and the comparative models, we performed cross-validation across the eight distinct dataset combinations. Specifically, each model was trained on one dataset combination and then tested on the remaining seven combinations. This approach ensures that the models are not overfitted to a single type of data configuration and can effectively generalize the learned features for motion direction detection. The rationale behind this cross-validation strategy is to assess whether each model can extract truly useful features from the two-channel images that generalize well across different background and object color configurations. By training and testing across varied combinations, we mitigate the risk of overfitting and gain insights into the models’ robustness and feature extraction capabilities. For each of the eight dataset combinations (DL, DR, LD, LL, LR, RD, RL, RR), we conducted the following steps: (1) Train the model using images from one specific combination. (2) Evaluate the model’s performance on the remaining seven combinations. (3) Record the accuracy for each test set to analyze the generalization performance. This comprehensive cross-validation allows us to quantify how well each model can handle diverse scenarios in terms of object size, color, and background configuration, ensuring a robust assessment of their motion direction detection capabilities. The cross-validation results are summarized in Table 1 and Table 2, providing detailed accuracy metrics for each model across all dataset combinations. This analysis not only highlights the strengths and weaknesses of the proposed model but also benchmarks its performance against established state-of-the-art models in terms of generalization ability.

In the simulations, we conducted multiple experiments with ConvNeXt on LL and LR to evaluate its learning accuracy. The results showed that ConvNeXt had a low success rate of only 10% on LR, meaning that only 10% of the *N* experiments met the expected success criteria. To maintain consistency with the results of other models, we calculated the mean and standard deviation of 10 successful cases and also reported the overall success and failure rates of it. However, on LL, ConvNeXt showed no signs of success in 50 experiments. We define ‘successful learning’ as achieving a training accuracy greater than 75% and maintaining an accuracy fluctuation within 0.1% for 100 epochs after reaching this accuracy. On LL, despite various optimization attempts (including adjusting the learning rate, changing the batch size, increasing L2 regularization, and extending the training epochs), ConvNeXt_Tiny consistently failed to meet this standard. Consequently, we recorded the results of 10 failed experiments and analyzed these results in detail. To improve the success rate of ConvNeXt on LL and LR, we tried various optimization methods, including adjusting the learning rate, changing the batch size, increasing L2 regularization, and extending the training epochs. However, these optimizations had minimal impact on the success rate of the model. On LL, the success rate of ConvNeXt remained around 10%, while on LR, ConvNeXt could not succeed at all. It is noteworthy that other models (AVS, Four-Layer CNN, and others) rarely failed in their experiments across all datasets, with only 1–2 errors occurring on very few datasets, which is entirely acceptable. In contrast, ConvNeXt had an extremely low success rate on LL and almost no success on LR. This suggests that the low success rate of ConvNeXt on these specific datasets may be related to the characteristics of the datasets rather than an inherent problem with the model itself because of its lack of multi-task function and hyper-complex structure. To ensure comprehensive and reliable results, we meticulously recorded and analyzed all experimental outcomes, listing each experiment’s results in the Data availability. Our study indicates that while ConvNeXt performs well on some datasets, it has an extremely low success rate on LL and LR. Since ConvNeXt is not the primary model we designed and aimed to validate, this discussion mainly serves to demonstrate the robustness of our own model across various datasets. There is no necessity to delve deeply into the reasons behind ConvNeXt’s inability to fit certain datasets, as our focus remains on verifying the performance and generalizability of our proposed AVS model.

Through cross-validation, we evaluated the generalization ability of the proposed model and the baseline models across eight different dataset combinations. Each model was trained on one dataset combination and then tested on the remaining seven combinations; the corresponding validation accuracy is colored yellow for clarification. This approach ensures that the models do not overfit a particular dataset configuration and can effectively generalize the learned features. The experimental results indicate that simpler models tend to perform worse in terms of generalization during cross-validation. But the AVS model achieved high training accuracy (close to 100%) across most training combinations and performed well on the corresponding test combinations. However, accuracy dropped significantly when tested on some combinations (e.g., RD and RL), indicating that these combinations present challenges. This result shows that some of the local features lead to easier ways to obtain local minima due to variations in background and object color. Specifically, datasets like RD and RL exhibited characteristics that made them more challenging for the model, suggesting that variations in background and object color can affect the model’s performance.

Four-layer CNN and LeNet simulations showed that simplified CNNs exhibited significantly lower train and test accuracy compared to the AVS model. Their performance was highly inconsistent across different test combinations, indicating weaker generalization ability and difficulty handling diverse backgrounds and color configurations. The very low accuracy of cross-validation, lower than 50%, suggests that the models tend to fail in learning the features. This inconsistency highlights the limitations of simpler CNN architectures in adapting to varied data and capturing complex features compared to more advanced models. This can be attributed to their weakness in capturing global features across the two-channel motion direction dataset; only simpler features can be caught due to their structure.

More complex models, like EfficientNet-B0 and ResNet-50, also showed good training accuracy but had similar performance drops on some test combinations. They demonstrated better global feature extraction capabilities compared to simpler models. However, this strength also posed a limitation on their ability to achieve correct classifications, as the complexity of these models sometimes hindered their performance on localized motion direction tasks. The fact is that these models easily capture the features of the corresponding dataset; the learning accuracy and validation accuracy are kept at a high level when less random pixels appear. These models have over 99% accuracy on the corresponding dataset. In particular, EfficientNet-B0 displayed very low accuracy on certain combinations, suggesting poor adaptability to specific configurations. These decreases appeared much more frequently than in AVS, showing that the local features stand in a more necessary place than global features in motion direction images. The actual movement of objects tends to be localized within specific parts of the images, especially for judgments on specific direction.

In conclusion, the AVS model demonstrated the most stable performance across various backgrounds and color configurations, though there is still room for improvement in some combinations. The simplified CNNs showed clear deficiencies in generalization, struggling to handle complex backgrounds and color configurations. EfficientNet-B0 and ResNet-50, despite performing well on some test combinations, showed significant performance drops on others, indicating the need for further optimization in their generalization ability. ConvNeXt, despite being a sophisticated model, was shown to be unsuitable for this classification problem. Its poor performance on the LL and LR datasets suggests that its complex structure might not align well with the task’s requirements. In contrast, our AVS model successfully combines the low learning cost of simpler models with the precise global classification abilities of more complex models. Additionally, AVS excels in extracting local features, leading to higher overall accuracy. Its ability to maximize local feature extraction on datasets rich in local information led to high accuracy across all datasets during cross-validation experiments. Overall, the AVS model outperformed the other models in terms of comprehensive performance across different dataset combinations. Its balance of simplicity, global feature extraction, and local feature precision highlights AVS as a robust model for motion direction detection, showcasing its adaptability and effectiveness across various dataset configurations.

### 3.4. Different Training Data Ratio

In this section, we aim to investigate the impact of varying the ratio between the training and testing datasets on the models’ ability to correctly identify motion direction. By modifying the training and testing split ratios while keeping the total data volume constant, we sought to understand each model’s robustness and generalization capabilities under conditions where training data were limited. Our dataset consisted of 10,000 randomly selected images from the aforementioned eight datasets. This selection was made to ensure a comprehensive representation of all object shapes, sizes, and colors in combination with different backgrounds. This approach maximizes the characteristics of the two-channel motion direction data, allowing the models to quickly learn and identify each type of feature. It also mitigates the risk of feature loss and overfitting associated with using a single dataset.

The experiment involved adjusting the training-to-testing data ratios to 75:25, 50:50, 10:90, and 5:95. For each ratio, we split the dataset according to the specified ratio. Then, we trained each model on the training portion of the dataset. Last, the evaluations of the model’s performance on the testing portion were recorded (see Table 3) to analyze the models’ performance. The variations in training-to-testing ratios allow us to observe how each model adapts to different amounts of training data and to assess their ability to generalize to unseen data under these conditions.

The AVS model demonstrated exceptional robustness across all training-to-testing ratios, maintaining near-perfect accuracy even with extremely limited training data. This performance is indicative of the model’s efficiency in leveraging the available training data to generalize well across different testing scenarios. This highlights the AVS model’s superior capability to generalize from small datasets. Four-Layer CNN and LeNet-5 showed a significant decline in performance as the training data decreased. With 75:25 and 50:50 splits, their test accuracies were moderate. But with more extreme splits (10:90 and 5:95), their test accuracies dropped drastically, indicating a weaker ability to generalize from limited training data. These results suggest that both models struggle to effectively utilize small amounts of training data, which may be due to their limited capacity to capture complex features necessary for accurate classification. EfficientNet-B0 and ResNet50 retained relatively high training accuracies but exhibited noticeable drops in test accuracy as the training data ratio decreased. EfficientNet-B0, despite its pre-training on ImageNet, performed better than ResNet50 on extreme splits but still showed reduced generalization capability compared to AVS. The performance drop in EfficientNet-B0 and ResNet50 on extreme splits highlights the challenge of achieving a balance between model complexity and generalization ability, especially when training data are severely limited. ConvNeXt struggled significantly, especially with reduced training data. The high variance in test accuracy for 75:25 and 50:50 splits and extremely low test accuracies for 10:90 and 5:95 splits indicate that ConvNeXt is less suitable for this classification task.

In conclusion, the AVS model consistently outperformed other models across various training-to-testing ratios, demonstrating its robustness and superior generalization capabilities. Simpler models showed a pronounced decline in performance, with less training data, while more complex models like EfficientNet-B0 and ResNet50 performed better but still could not match the AVS model’s efficacy. ConvNeXt’s poor performance suggests that its complexity may be a disadvantage for this specific task.

### 3.5. Noise-Immunity Simulation

In this section, we assessed the noise immunity of the models trained with various training-to-testing ratios by introducing salt-and-pepper noise to the dataset. Salt-and-pepper noise is characterized by the presence of randomly distributed black and white pixels, which simulate common types of image noise encountered in real-world scenarios, such as sensor noise or transmission errors. We chose to use salt-and-pepper noise for validation, since it offers realism, simplicity, and challenging conditions. It closely mimics noise patterns found in real-world applications, providing a realistic evaluation of the models’ robustness. The random distribution of noise makes it a straightforward method to implement and analyze. Furthermore, this type of noise introduces significant disturbances to the image, challenging the models to maintain accuracy despite the noise, thereby testing their ability to generalize and recognize underlying patterns. To conduct this experiment, we applied four different levels of salt-and-pepper noise to the dataset: 1%, 2%, 5%, and 10% of the total pixels. The sum of black and white noise pixels matched these percentages, but the exact numbers of black and white pixels were randomly distributed across the images.

We evaluated the performance of each model, trained with different training data ratios, on the noise-augmented datasets. The results of this noise-immunity simulation are presented in Table 4. This evaluation helps us understand how well the models can retain their accuracy when exposed to noisy data and highlights their robustness and reliability in practical applications.

In our noise-immunity simulation, we observed distinct differences in the performance of the models when exposed to increasing levels of salt-and-pepper noise. The AVS model consistently outperformed the other models across all noise levels and training-to-testing ratios. Specifically, it maintained high accuracy even with 10% noise, demonstrating robust noise immunity. The performance of other models deteriorated significantly as noise levels increased, with the AVS model showing a clear advantage in handling noise. At higher noise levels, the accuracy of the simpler models like Four-Layer CNN and LeNet-5 dropped sharply, indicating their limited ability to generalize under noisy conditions. In contrast, more complex models like EfficientNet-B0 and ResNet-50, while performing better than simpler models, still showed significant degradation in accuracy under high noise conditions. ConvNeXt also struggled with noise, particularly at higher noise levels and lower training-to-testing ratios.

Overall, the AVS model demonstrated superior robustness and reliability in practical applications where noise was prevalent, making it a preferable choice for motion direction detection tasks in noisy environments.

### 3.6. Simulation on Real-World and Rotated MNIST Datasets

To address the possibilities of the limited scope of the dataset used in previous experiments, we conducted additional evaluations using more complex and diverse real-world datasets and the rotated MNIST dataset, both of which were tailored to test motion direction detection capabilities.

**Figure 8:**
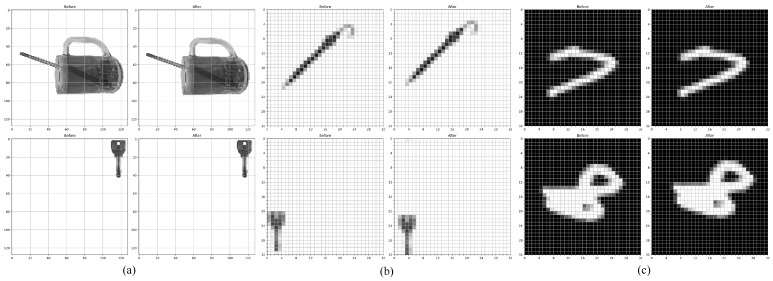
Samples of narrowed real objects and moving rotated MNIST dataset images and dataset generation. (**a**) shows samples for a ’real object’ moving ’left-down’ and ’left’; (**b**) shows samples for a ’narrowed real object’ moving ’left-up’ and ’right-down’; (**c**) shows samples for a ’rotated MNIST’ moving ’right’ and ’right-up’.

For the real-world dataset, we captured images using an iPhone and converted them to single-channel grayscale images after removing their backgrounds. The images are like the ’Before’ image in Figure 8a. The objects were placed at fixed angles to generate a static dataset of real-world objects. Given that simpler convolutional neural networks (CNNs), such as the Four-Layer CNN and LeNet-5, struggle with varying image sizes, we created a narrowed dataset where these real-world object images were downscaled to 32×32 pixels, as in the ’Before’ image in Figure 8b. This ensured the preservation of objects’ shape and color features while only reducing image clarity. We then generated a motion direction dataset by moving these downscaled images by one pixel in specified directions and labeling them accordingly, as illustrated in Figure 8a,b.

For the rotated MNIST dataset, the original 28×28 grayscale images were padded to 32×32 pixels by adding background pixels to the periphery. To enhance the dataset’s robustness, we applied data augmentation by rotating each digit at various angles, creating a static rotated MNIST dataset. Subsequently, we created a dynamic version of this dataset by shifting the images by one pixel in a specific direction, similar to real-world dataset processing, as depicted in Figure 8c.

We used a 75:25 split for training and testing, ensuring that the models were trained and evaluated under a wide range of conditions, including mixed backgrounds and varying object orientations. The results, as summarized in Table 5, demonstrate that our AVS model outperforms other vision processing models on these datasets. Specifically, our AVS achieved a perfect 100.00% accuracy on both the narrowed real-world object and rotated MNIST datasets. This indicates that our model can accurately capture and process motion direction features in complex, real-world scenarios.

The superior performance of AVS in comparison to models like EfficientNet-B0 (EfNB0) and ResNet50, which also showed reasonable success, highlights the effectiveness of our model in learning motion direction features rather than merely fitting simple datasets. The discrepancies between EfNB0 and ResNet on these tasks further suggest that while these complex models have learned motion direction features, their success is not purely due to random fitting of simplified datasets. This validates the robustness and generalizability of our AVS in handling diverse and real-world image datasets.

## 4. Conclusions and Discussion

In this study, we proposed a learning motion direction detection model for grayscale images using the dendritic neuron model, incorporating dendrites into the detection mechanism. We introduced a novel learning AVS for two-dimensional global motion direction detection, inspired by biological motion detection mechanisms. Our approach relies on local motion direction information obtained from local motion direction detective neurons to infer global motion direction, and this detection ability can be learned. By convolving over two-dimensional images before and after motion, we trained our model to learn global motion direction detection in grayscale images. The proposed neuron model aligns with the visual mechanisms of living organisms, such as photoreceptor cells, horizontal cells, and ganglion cells in the retina. Our results demonstrated that the proposed learning AVS effectively acquired visual motion direction detection capabilities for grayscale images, successfully predicting the shapes of local motion direction detective neurons, similar to the Barlow and Levick model. The model showed high accuracy in detecting motion direction in noise-free grayscale images and acquired shapes corresponding to motion direction detection. Testing with datasets for testing indicated strong versatility, unaffected by grayscale values of objects and backgrounds or object size in other positions. This indicates that our model, even when trained on datasets with a single feature, achieves high accuracy in most cases. While the accuracy of our model decreased in noisy datasets, it remained much higher than that of CNNs, showcasing its significant noise resistance. The proposed learning AVS possesses many desirable properties useful in other visual perception systems, potentially being an integral part of the human visual system. It provides new insights into how visual input is processed at different stages of the visual system.

Although supervised learning using backpropagation is utilized, the exact learning mechanism of neurons in living organisms remains unclear. It is believed that learning occurs via mechanisms akin to unsupervised learning, such as competitive learning using Hebb’s rule, suggesting that a model incorporating this would be more appropriate [48]. We also believe that detecting color images is feasible using the current mechanism because color images can be converted to grayscale by averaging or using weighted sum of the RGB (red, green, blue) channels or treating each of the three RGB channels as an independent feature map and processing it separately with the system. Despite being a simplified model not accounting for the detailed functioning of the visual system and brain mechanisms, it offers a quantitative explanation for many known neurobiological visual phenomena. The learning AVS can be easily applied to other visual perceptions, such as orientation direction, motion speed, and binocular vision perception, and even to other sensory systems like olfaction, taste, and touch. Therefore, we hope that the model proposed in this paper will contribute to elucidating the visual mechanisms of living organisms and advancing research into the detection of object movement direction.

The proposed model has potential applications in various real-world scenarios, such as helping self-driving cars detect and predict the direction of other vehicles, pedestrians, and cyclists, thereby improving collision avoidance and overall safety. However, the model has some limitations and and restrictions. Although the model is based on biologically inspired designs, it may still have significant differences from real biological systems. In particular, the dendritic neuron model used in this study may not fully replicate the nonlinear interactions observed in real compartmentalized neuron models. Previous research has shown that biological neurons exhibit more complex behaviors due to the intricate structure and interactions within dendritic compartments [49,50]. Future research could focus on further integrating these biological behaviors to enhance the model’s performance. Our model has demonstrated superior performance in motion direction detection compared to CNNs. However, to fully establish its versatility robustness and generality, it also must be challenged with tasks such as image classification, object detection, and image segmentation.

## Figures and Tables

**Table 1:** Cross-validation of different datasets (simplified models). (The yellow boxes mean the training dataset and test dataset come from the same kind of images).

Model	Train Data	Train Acc	Test Acc							
			DL	DR	LD	LL	LR	RD	RL	RR
AVS	**DL**	99.71 ± 0.12%	99.61 ± 0.21%	100.0 ± 0.00%	99.57 ± 0.25%	99.67 ± 0.25%	100.0 ± 0.00%	74.08 ± 2.07%	73.63 ± 1.87%	80.19 ± 1.01%
	**DR**	100.0 ± 0.00%	99.96 ± 0.04%	100.0 ± 0.00%	99.99 ± 0.02%	99.99 ± 0.02%	100.0 ± 0.00%	16.99 ± 5.32%	16.93 ± 5.72%	41.09 ± 5.94%
	**LD**	99.73 ± 0.09%	99.73 ± 0.14%	100.0 ± 0.00%	99.71 ± 0.15%	99.69 ± 0.24%	99.99 ± 0.01%	74.23 ± 2.54%	73.58 ± 3.46%	80.78 ± 1.98%
	**LL**	9.70 ± 0.20%	99.66 ± 0.17%	100.0 ± 0.00%	99.62 ± 0.20%	99.62 ± 0.19%	100.0 ± 0.00%	74.23 ± 3.01%	73.39 ± 3.62%	80.69 ± 3.26%
	**LR**	100.0 ± 0.00%	99.96 ± 0.12%	100.0 ± 0.00%	99.98 ± 0.08%	99.96 ± 0.06%	100.0 ± 0.00%	15.52 ± 4.29%	15.85 ± 3.96%	39.31 ± 3.80%
	**RD**	99.58 ± 0.08%	32.09 ± 8.30%	44.87 ± 5.68%	31.87 ± 7.30%	31.31 ± 6.67%	45.57 ± 5.95%	99.56 ± 0.14%	99.26 ± 0.13%	99.54 ± 0.20%
	**RL**	99.25 ± 0.06%	34.09 ± 6.92%	46.48 ± 6.99%	32.97 ± 8.93%	33.13 ± 10.42%	47.10 ± 6.51%	99.59 ± 0.17%	99.27 ± 0.19%	99.53 ± 2.15%
	**RR**	99.63 ± 0.05%	64.41 ± 29.65%	86.16 ± 14.29%	64.69 ± 29.81%	64.45 ± 30.16%	86.33 ± 14.34%	93.68 ± 3.01%	92.60 ± 3.16%	99.54 ± 0.18%
Four-Layer CNN	**DL**	97.67 ± 0.29%	96.26 ± 0.63%	96.58 ± 0.47%	41.13 ± 15.49%	29.33 ± 5.52%	36.06 ± 10.57%	17.63 ± 3.22%	12.85 ± 1.71%	12.96 ± 2.41%
	**DR**	97.61 ± 0.15%	95.27 ± 1.00%	96.06 ± 0.64%	26.98 ± 13.01%	24.34 ± 7.02%	29.52 ± 9.45%	17.13 ± 3.27%	12.91 ± 1.24%	14.16 ± 2.16%
	**LD**	96.46 ± 0.76%	39.04 ± 11.37%	38.51 ± 11.74%	94.08 ± 0.13%	54.73 ± 5.63%	66.15 ± 6.39%	15.18 ± 3.26%	12.65 ± 2.70%	14.10 ± 2.54%
	**LL**	93.60 ± 2.23%	91.69 ± 1.92%	91.57 ± 1.78%	92.32 ± 1.70%	87.43 ± 3.07%	89.58 ± 2.00%	19.99 ± 3.11%	14.51 ± 2.16%	14.87 ± 2.17%
	**LR**	95.00 ± 1.72%	88.42 ± 2.63%	89.31 ± 2.62%	88.99 ± 3.04%	86.43 ± 3.47%	90.74 ± 1.88%	16.10 ± 2.58%	13.87 ± 3.41%	15.42 ± 2.41%
	**RD**	100.0 ± 0.00%	6.41 ± 3.97%	4.11 ± 2.61%	85.85 ± 4.95%	37.16 ± 4.99%	37.21 ± 5.03%	73.61 ± 1.84%	23.61 ± 2.34%	26.74 ± 3.27%
	**RL**	79.23 ± 3.15%	16.94 ± 3.38%	16.38 ± 3.90%	17.09 ± 4.96%	15.01 ± 2.19%	14.84 ± 3.29%	16.34 ± 3.42%	12.90 ± 1.34%	13.43 ± 1.36%
	**RR**	88.97 ± 3.15%	33.45 ± 11.09%	32.99 ± 11.80%	32.89 ± 12.10%	23.29 ± 5.65%	31.10 ± 9.53%	25.03 ± 5.75%	17.98 ± 3.39%	26.56 ± 5.93%
LeNet	**DL**	97.05 ± 4.65%	96.27 ± 4.72%	96.16 ± 5.33%	26.65 ± 11.20%	21.55 ± 4.26%	24.65 ± 7.64%	18.64 ± 6.84%	12.76 ± 2.15%	12.75 ± 1.05%
	**DR**	98.21 ± 3.67%	96.72 ± 3.98%	97.76 ± 3.31%	33.37 ± 3.90%	20.68 ± 2.71%	23.59 ± 3.35%	23.07 ± 4.67%	13.71 ± 1.68%	13.38 ± 1.14%
	**LD**	92.94 ± 5.17%	34.54 ± 21.48%	32.61 ± 19.46%	89.21 ± 6.35%	51.01 ± 7.27%	60.21 ± 8.15%	17.18 ± 2.42%	13.00 ± 1.59%	15.15 ± 2.07%
	**LL**	95.18 ± 3.53%	88.96 ± 3.73%	89.11 ± 3.03%	89.00 ± 3.60%	88.41 ± 3.31%	86.80 ± 3.84%	16.92 ± 2.82%	12.96 ± 1.99%	13.92 ± 1.43%
	**LR**	93.89 ± 5.72%	88.08 ± 6.69%	89.45 ± 6.86%	89.62 ± 5.54%	86.93 ± 4.38%	90.67 ± 4.96%	14.85 ± 2.80%	14.44 ± 2.47%	14.63 ± 3.58%
	**RD**	95.78 ± 2.28%	45.29 ± 6.73%	38.23 ± 6.30%	74.59 ± 6.90%	26.39 ± 4.72%	36.29 ± 6.28%	67.91 ± 4.92%	21.39 ± 3.05%	28.09 ± 4.27%
	**RL**	79.34 ± 7.47%	12.96 ± 1.52%	12.38 ± 0.83%	11.84 ± 1.77%	12.09 ± 1.32%	11.98 ± 0.71%	12.69 ± 0.70%	12.83 ± 0.69%	12.44 ± 0.65%
	**RR**	77.82 ± 7.39%	12.52 ± 0.79%	13.01 ± 0.73%	12.63 ± 1.09%	13.33 ± 1.05%	13.40 ± 1.45%	12.50 ± 0.51%	12.66 ± 0.70%	12.64 ± 0.73%

**Table 2:** Cross-validation of different datasets (improved models). (The yellow boxes mean the training dataset and test dataset come from the same kind of images).

Model	Train Data	Train Acc	Test Acc							
			DL	DR	LD	LL	LR	RD	RL	RR
Efficien tNet-B0	**DL**	99.79 ± 0.03%	99.30 ± 0.18%	99.71 ± 1.77%	22.79 ± 3.64%	21.94 ± 2.73%	25.38 ± 3.47%	13.11 ± 1.74%	12.85 ± 0.92%	12.01 ± 1.76%
	**DR**	99.84 ± 0.05%	98.01 ± 0.40%	99.49 ± 0.16%	22.39 ± 2.87%	20.66 ± 2.26%	22.82 ± 2.97%	12.59 ± 3.07%	12.46 ± 2.91%	12.94 ± 1.59%
	**LD**	99.62 ± 0.12%	15.73 ± 8.27%	14.91 ± 7.37%	98.99 ± 0.23%	57.40 ± 5.26%	60.30 ± 4.53%	16.16 ± 4.15%	14.10 ± 2.30%	12.99 ± 1.82%
	**LL**	99.42 ± 0.08%	97.84 ± 0.42%	98.61 ± 0.26%	97.89 ± 0.61%	97.85 ± 0.54%	96.43 ± 1.29%	14.89 ± 1.95%	11.74 ± 2.49%	12.89 ± 2.36%
	**LR**	99.64 ± 0.08%	95.89 ± 3.30%	98.44 ± 4.18%	95.77 ± 6.95%	95.22 ± 6.73%	98.11 ± 0.24%	12.27 ± 2.21%	12.61 ± 2.05%	12.99 ± 2.52%
	**RD**	99.97 ± 0.03%	12.67 ± 2.70%	13.52 ± 2.46%	14.83 ± 3.74%	13.79 ± 2.02%	14.08 ± 2.37%	84.15 ± 1.05%	25.42 ± 1.32%	23.71 ± 1.71%
	**RL**	99.93 ± 0.03%	18.24 ± 1.62%	18.74 ± 3.91%	15.75 ± 3.20%	15.64 ± 2.34%	15.30 ± 2.52%	37.60 ± 1.74%	23.59 ± 0.86%	16.91 ± 1.59%
	**RR**	99.93 ± 0.03%	13.04 ± 1.83%	14.00 ± 2.00%	13.30 ± 1.99%	11.80 ± 1.51%	12.87 ± 1.06%	23.46 ± 3.01%	19.58 ± 1.24%	31.05 ± 0.02%
ResNet 50	**DL**	99.99 ± 0.01%	99.96 ± 0.04%	99.94 ± 0.07%	12.25 ± 1.92%	17.06 ± 2.03%	18.82 ± 1.85%	12.61 ± 1.49%	12.43 ± 1.75%	13.80 ± 2.63%
	**DR**	99.94 ± 0.04%	99.39 ± 0.24%	99.85 ± 0.08%	13.07 ± 1.47%	18.63 ± 2.17%	21.19 ± 4.12%	11.68 ± 1.29%	12.53 ± 1.61%	11.56 ± 2.17%
	**LD**	99.89 ± 0.06%	7.67 ± 3.78%	7.59 ± 4.21%	99.96 ± 0.04%	46.91 ± 2.18%	50.87 ± 2.74%	17.17 ± 4.63%	13.53 ± 1.92%	12.81 ± 2.09%
	**LL**	99.81 ± 0.08%	98.32 ± 0.89%	99.31 ± 0.44%	98.03 ± 1.37%	97.35 ± 1.05%	98.21 ± 1.48%	14.47 ± 2.15%	14.06 ± 2.82%	13.36 ± 1.76%
	**LR**	99.85 ± 0.07%	96.79 ± 0.83%	99.32 ± 0.25%	97.18 ± 0.59%	96.56 ± 0.62%	99.10 ± 0.18%	16.57 ± 4.22%	13.49 ± 1.48%	15.36 ± 4.16%
	**RD**	100.0 ± 0.00%	13.15 ± 2.64%	12.54 ± 2.05%	12.98 ± 0.69%	13.40 ± 1.92%	12.66 ± 1.56%	13.88 ± 1.12%	12.77 ± 0.82%	12.69 ± 1.08%
	**RL**	100.0 ± 0.00%	13.71 ± 1.50%	12.59 ± 1.87%	12.51 ± 2.13%	13.30 ± 1.42%	12.04 ± 1.49%	12.07 ± 1.06%	12.56 ± 0.91%	12.53 ± 0.83%
	**RR**	100.0 ± 0.00%	12.43 ± 1.53%	12.68 ± 2.30%	11.84 ± 1.39%	12.66 ± 1.80%	13.21 ± 1.40%	12.47 ± 0.81%	12.44 ± 0.97%	12.59 ± 0.69%
Conv NeXt	**DL**	99.95 ± 0.04%	99.86 ± 0.10%	98.35 ± 0.26%	14.03 ± 4.70%	12.44 ± 1.64%	13.10 ± 1.54%	14.26 ± 2.60%	12.73 ± 1.68%	11.84 ± 0.94%
	**DR**	99.95 ± 0.05%	99.91 ± 0.08%	99.00 ± 0.33%	12.29 ± 2.12%	12.49 ± 1.97%	13.25 ± 1.40%	11.81 ± 2.82%	11.59 ± 1.84%	11.89 ± 2.06%
	**LD**	99.96 ± 0.02%	35.46 ± 14.22%	30.46 ± 13.44%	99.86 ± 0.11%	47.23 ± 3.67%	47.43 ± 1.29%	41.88 ± 8.59%	19.80 ± 2.69%	13.40 ± 0.98%
	**LL**	13.81 ± 0.53%	13.05 ± 2.51%	13.25 ± 2.59%	13.31 ± 3.42%	12.25 ± 1.83%	11.15 ± 1.63%	12.40 ± 1.39%	12.25 ± 2.54%	11.50 ± 2.54%
	**LR**	97.53 ± 1.90%	83.85 ± 22.15%	82.34 ± 20.66%	79.20 ± 23.54%	69.79 ± 23.98%	57.23 ± 15.17%	33.21 ± 9.58%	19.29 ± 2.87%	18.08 ± 2.62%
	**RD**	96.32 ± 0.88%	14.17 ± 2.03%	13.04 ± 2.85%	81.69 ± 9.32%	33.64 ± 5.72%	32.88 ± 5.95%	65.11 ± 6.69%	22.78 ± 2.19%	15.23 ± 1.80%
	**RL**	60.50 ± 25.68%	13.24 ± 1.90%	13.23 ± 1.90%	12.29 ± 1.57%	13.18 ± 1.48%	13.53 ± 1.83%	12.57 ± 0.79%	12.81 ± 0.71%	12.46 ± 0.65%
	**RR**	98.30 ± 0.97%	12.84 ± 1.10%	13.34 ± 1.67%	12.59 ± 1.43%	12.60 ± 0.97%	12.79 ± 1.67%	13.49 ± 0.94%	12.71 ± 1.02%	12.31 ± 0.47%

**Table 3:** Comparison of AVS with other vision processing systems on different training data and testing data ratios.

Model	Train Acc	Test Acc	Train Acc	Test Acc
Train–Test	75:25		50:50	
**AVS**	99.77 ± 0.18%	99.73 ± 0.21%	99.83 ± 0.04%	99.88 ± 0.06%
Four-Layer CNN	97.65 ± 0.45%	64.58 ± 2.55%	86.78 ± 6.76%	55.42 ± 4.11%
LeNet-5	88.69 ± 1.80%	51.91 ± 5.11%	91.09 ± 1.79%	47.23 ± 4.14%
EfficientNet-B0	99.85 ± 0.61%	76.49 ± 2.85%	98.74 ± 1.08%	72.73 ± 3.07%
ResNet50	99.93 ± 0.06%	70.10 ± 2.64%	99.57 ± 0.29%	66.33 ± 6.26%
ConvNeXt	98.23 ± 4.44%	46.36 ± 17.2%	97.17 ± 4.33%	41.46 ± 19.6%
Train–Test	10:90		5:95	
**AVS**	99.79 ± 0.16%	99.70 ± 0.31%	99.52 ± 0.51%	99.30 ± 0.72%
Four-Layer CNN	92.35 ± 3.05%	20.06 ± 2.47%	93.89 ± 3.02%	17.10 ± 2.17%
LeNet-5	92.45 ± 1.35%	14.71 ± 5.98%	93.64 ± 1.70%	13.50 ± 0.69%
EfficientNet-B0	98.67 ± 0.58%	53.84 ± 2.75%	98.16 ± 1.14%	32.38 ± 3.90%
ResNet50	100.0 ± 0.00%	12.63 ± 1.00%	98.09 ± 2.28%	12.17 ± 0.48%
ConvNeXt	99.12 ± 1.42%	13.88 ± 0.66%	99.90 ± 0.25%	12.69 ± 0.95%

**Table 4:** Comparison of AVS with other vision processing systems on noise immunity.

Noises	1%	2%	5%	10%
Train–Test	75:25			
**AVS**	99.51 ± 0.12%	98.89 ± 0.27%	95.43 ± 2.65%	89.03 ± 4.54%
Four-Layer CNN	45.62 ± 4.05%	39.92 ± 3.82%	33.58 ± 3.85%	28.53 ± 3.69%
LeNet-5	35.19 ± 2.68%	30.06 ± 2.96%	23.81 ± 2.59%	20.39 ± 2.74%
EfficientNet-B0	67.14 ± 2.08%	61.84 ± 2.66%	53.36 ± 3.29%	46.54 ± 2.31%
ResNet50	47.73 ± 4.55%	43.39 ± 3.17%	37.42 ± 2.08%	30.74 ± 1.83%
ConvNeXt	40.98 ± 13.44%	37.89 ± 10.00%	31.87 ± 9.48%	27.40 ± 8.11%
Train–Test	50:50			
**AVS**	99.54 ± 0.19%	99.08 ± 0.28%	97.79 ± 0.95%	94.24 ± 1.74%
Four-Layer CNN	39.06 ± 3.62%	34.66 ± 2.84%	29.18 ± 2.70%	24.29 ± 2.78%
LeNet-5	31.87 ± 3.03%	27.34 ± 2.88%	22.63 ± 2.14%	18.70 ± 1.08%
EfficientNet-B0	58.29 ± 4.57%	53.72 ± 3.25%	47.09 ± 3.02%	40.34 ± 2.18%
ResNet50	46.46 ± 5.25%	41.39 ± 4.27%	35.07 ± 4.87%	27.82 ± 3.37%
ConvNeXt	35.09 ± 16.79%	31.86 ± 15.46%	26.84 ± 10.89%	23.09 ± 9.68%
Train–Test	10:90			
**AVS**	99.24 ± 0.47%	98.03 ± 1.65%	93.90 ± 3.60%	86.86 ± 4.38%
Four-Layer CNN	16.92 ± 1.48%	16.23 ± 0.96%	14.89 ± 1.26%	13.98 ± 1.12%
LeNet-5	13.90 ± 0.92%	13.03 ± 0.72%	13.09 ± 0.91%	12.67 ± 0.85%
EfficientNet-B0	33.83 ± 3.92%	29.39 ± 4.10%	24.69 ± 3.14%	21.06 ± 1.89%
ResNet50	13.04 ± 0.75%	12.79 ± 1.01%	12.57 ± 0.88%	12.75 ± 0.47%
ConvNeXt	13.36 ± 1.18%	13.28 ± 0.40%	13.24 ± 0.80%	12.71 ± 0.94%
Train–Test	5:95			
**AVS**	99.04 ± 0.55%	95.08 ± 6.64%	93.26 ± 3.92%	87.46 ± 6.39%
Four-Layer CNN	14.83 ± 2.27%	14.79 ± 1.49%	14.13 ± 1.54%	13.88 ± 1.15%
LeNet-5	12.48 ± 1.35%	12.44 ± 0.84%	12.37 ± 0.74%	12.59 ± 0.69%
EfficientNet-B0	23.40 ± 2.39%	20.81 ± 2.28%	17.66 ± 1.41%	16.04 ± 1.39%
ResNet50	12.64 ± 0.51%	12.60 ± 0.95%	12.23 ± 0.85%	12.78 ± 0.71%
ConvNeXt	13.11 ± 0.93%	12.84 ± 1.21%	13.09 ± 0.82%	12.48 ± 0.86%

**Table 5:** Comparison of AVS with other vision processing systems on other datasets.

Dataset	Real Object	Narrowed Real Object	Rotated MNIST
**AVS**	99.90 ± 0.21%	100.00 ± 0.00%	100.00 ± 0.00%
Four-Layer CNN	None	44.80 ± 5.91%	68.65 ± 7.16%
LeNet-5	None	35.65 ± 7.75%	66.45 ± 9.24%
EfficientNet-B0	57.30 ± 12.52%	78.80 ± 12.04%	78.70 ± 8.62%
ResNet50	62.85 ± 6.25%	67.45 ± 8.27%	89.19 ± 10.46%
ConvNeXt	36.05 ± 12.31%	58.05 ± 21.41%	67.45 ± 16.32%

## Data Availability

The data used in this study are limited-access but available upon reasonable request. Interested parties may request access by contacting corresponding author at yktodo@se.kanazawa-u.ac.jp. Access is subject to approval and compliance with confidentiality and ethical guidelines. The authors are committed to facilitating access within legal and ethical boundaries. https://wandb.ai/ruriiiii/GS_Motion_Simulation?nw=nwuserruriiiii (accessed on 9 July 2024) and https://wandb.ai/ruriiiii/GS_Motion_Validation?nw=nwuserruriiiii (accessed on 10 August 2024) are available upon reasonable request. Dendritic neuron model-based https://github.com/RurIIIIIkitesumimasen/Dendritic_GS_Motion (accessed on 11 August 2024) are available.
